# Central European journal of operations research (CJOR) “operations research applied to health services (ORAHS) in Europe: general trends and ORAHS 2020 conference in Vienna, Austria”

**DOI:** 10.1007/s10100-021-00792-z

**Published:** 2021-12-10

**Authors:** Roberto Aringhieri, Patrick Hirsch, Marion S. Rauner, Melanie Reuter-Oppermanns, Margit Sommersguter-Reichmann

**Affiliations:** 1 Dipartimento di Informatica, Università degli Studi di Torino, Corso Svizzera 185, 10149 Torino, Italy; 2 Institute of Production and Logistics, University of Natural Resources and Life Sciences, Feistmantelstraße 4, 1180 Vienna, Austria; 3 School of Business, Economics, and Statistics, Department of Business Decisions and Analytics, University of Vienna, Oskar-Morgenstern-Platz 1, 1090 Vienna, Austria; 4 Department of Law and Economics, Information Systems, Software and Digital Business Group, Technical University of Darmstadt, Hochschulstr. 1, 64289 Darmstadt, Germany; 5 School of Business, Economics, and Social Sciences, Department of Finance, Karl-Franzens University Graz, Universitaetsstraße 15, Resowi G2, 8010 Graz, Austria

**Keywords:** Strategic health care management, Health economics, Health care workforce scheduling, Health care operations management, Disease policy modelling, Emergency medical services, 90-02

## Abstract

This articles provides a short summary of the research topics and latest research results of the European Working Group “Operations Research Applied to Health Services” (ORAHS) organized as an e-conference in Juli 2020 at the University of Vienna, Austria (https://orahs2020.univie.ac.at/). Furthermore, challenges for OR in health care including application areas, decision support systems, general trends, and modelling techniques are briefly illustrated from an European and international perspective by providing selected essential literature reviews.

## Introduction

Health care around the world is facing unprecedented challenges due to global development. There are ongoing technological advances in health care but they are not affordable for everyone (Amiri et al. 2021). Migration and displacement pose one of the most significant humanitarian challenges (Bozorgmehr et al. 2020). The changing population structure in many developed countries requires new approaches and solutions for health care (Rouzet et al. 2019). The slowdown in economic development in some countries around the world is also putting public health budgets under pressure (Karanikolos et al. 2016). Climate change and political instability are increasingly affecting the health sector (Watts et al. 2020). As epidemics such as the current corona virus disease (COVID)-19 as well as various natural and man-made disasters pose particular threats, the health care sector must prepare for them urgently (e.g., Sharma et al. 2021; Makwana 2020). Health care policy makers are therefore forced to provide high quality services with tight budgets and a limited workforce by taking ethical issues into account. For this, policy makers need decision support by qualitative and quantitative Operations Research (OR) approaches as well as decision support systems (DSS) in order to prepare for these challenging situations by providing solutions for simple to even extreme scenarios.

To cope with health care challenges, the **EURO Working Group on Operational Research Applied to Health Services (ORAHS)** was formed in 1975 as part of a program for developing special interest groups within the European branch, EURO, of the International Federation of Operational Research Societies, IFORS (http://orahs.di.unito.it/about.html). The group has currently more than 330 members from more than 30 countries, mainly from Europe but also from overseas (e.g., USA, Canada, and Australia). The group meets for a week each summer in a different, mainly European, host country to discuss 140 talks and tens of posters on average. The objectives of the group are to impart ideas, knowledge and experience on the application of OR approaches and methods to problems in the health services area, mutual support of members, co-operation on joint projects and stimulation of approaches and attitudes in the field of applied Operational Research.

The ORAHS group has been very active which is also represented by several special issues in international scientific journals related to the yearly meetings (e.g., Rauner and Vissers 2003, Davies and Bensley 2005, Brailsford and Harper 2007, Dexter 2009, Xie et al. 2010, Aringhieri et al. 2013, 2016a, 2016b; Hans and Vliegen 2014, Çayırlı 2015, Mallor et al. 2018) as well as special issues and review papers on general trends in applications of OR in the health care sector (e.g., Rauner et al. 2005, Harper and Baker 2005, Brailsford and Harper 2008, Brailsford et al. 2009, Rais and Viana 2010, Brailsford and Vissers 2011, Brailsford et al. 2012, Weber et al. 2014, Morton et al. 2016, Royston 2016, Morton et al. 2021). Furthermore, several books edited by group members appeared over the last 20 years (e.g., Brandeau et al. 2004, Vissers and Beech 2005, Ozcan 2005, Zaric 2013, Brailsford et al. 2014, Carter et al. 2018, Kahraman and Topcu 2018, Masmoudi et al. 2021). Furthermore, the group members are actively involed in editorial boards of national and international OR societies. Finally, journals were established by efforts of our members such as Operation Research for Health Care (Elsevier), Health Systems (Taylor & Francis), and Health Care Management Science (Springer).

In 2020, the 46th meeting was virtually organized as a zoom conference at the University of Vienna, Austria (https://orahs2020.univie.ac.at/) due to the COVID(corona virus disease)-19 pandemic. More than 150 participates contributed with more than 100 scientific papers including two outstanding keynotes, a round table on prevention, a COVID-19 policy modelling session, and two sessions of the working group on health care and disaster management of the Austrian OR Society (ÖGOR), https://oegor-hcdm.univie.ac.at/.

This issue of the Central European Journal or Operations Research reflects some selected contributions on key health care policy problems that cover a variety of applicaton areas, topics, and modelling techniques applied from the members of ORAHS presented/discussed at this meeting as exemplarily outlined in Sects. 2 to 6. We furthermore briefly outline trends for further research in this area.

## Strategic health care policy making

Strategic health care management covers a wide range of application areas such as the establishment of health care systems/networks, as well as evaluation of health care programs and preventions strategies in which OR policy modelling plays an important role as illustrated at ORAHS 2020 (https://orahs2020.univie.ac.at/).

In the session on health care networks at ORAHS 2020, Brailsford et al. (2020a) outlined the importance of OR methods in decision making in health care and social care by overcoming barriers of implementation illustrated on a case study from the National Health Service in the United Kingdom. Tuson et al. (2020) assessed an Adverse Childhood Experiences program for Public Health Wales by using simulation and statistical analysis. Aspland et al. (2020) introduced to Sim.Pro.Model which is a useful tool for modelling, simulating, and visualizing clinical pathways as shown on a case study for supporting decision making at a Walsh cancer center. Vasilakis and Wood (2020) developed an open-source simulation tool to account for the requirements of end-users to help overcome poor uptake of OR in health care illustrated on the centralization of an acute stroke pathway.

Next, in the session on behavioral OR & policies in health care at ORAHS 2020, Oliveira et al. (2020) showed how important it is to involve stakeholvers to develop OR tools for better health care policy making by using the Delphi method. Kazakov et al. (2020) investigated external reference pricing methods to improve drug access, affordability, and availability in the EU by using a hybrid agent-based and system dynamics simulation model to accont for the behavoir of essential market actors. Barrera Ferro et al. (2020) applied a Health Belief Model to explore the reasons for no-show behaviour in a cervical cancer screening program among low-income patients in Bogotá, Colombia to further improve an outreach program by using simulation.

In the session on health care modelling in developing countries at ORAHS 2020, Duque-Uribe et al. (2020) explored rules for advancing sustanibility of supply chain management in hospitals. Chandra and Vipin (2020) analyzed well-performing supply contracts for a better provision of a rota virus immunization program in India by using mathematical models and expert opinions. Potgieter and Matter (2020) reported on improving epidemic response in the context of rural insufficient and unstable road infrastructure using a disease policy model and resource allocation techniques.

Furthermore, in the session on machine learning approaches applied to health care to better plan for and improve patient treatment at ORAHS 2020, interesting case studies were presented by colleagues from all over the world such as Koc University, Turkey (Demiray et al. 2020), Laboratoire Génie Industriel, CentraleSupelec, Gif-sur-Yvette, France (Santamaria-Acevedo 2020), and Stanford University, USA (Brandeau 2020).

Finally, one of the highlights of the e-conference was the “Round Table on Challenges in Health Care Prevention” which was moderated by Prof. Bernhard Schwarz from Medical University of Vienna, Austria who is also the head of the Karl Landsteiner Society, Austria. The further panalists included: Sally Brailsford (University of Southampton, UK), Margaret Brenadeau (Stanford University, USA), and Alexandra Schosser (BBRZ Med GmbH, Austria). They lively discussed areas of general prevention strategies, cancer prevention, psychiatric prevention, as well as COVID-19 prevention strategies with the e-audience.

In this special issue of CJOR, an health economic study entitled “The Benefit of an Ambulant Psychiatric Rehabilitation Program in Vienna, Austria: an Uncontrolled Repeated Measures Study” (Schosser et al. 2021) is included which was briefly presented in the round table session on prevention. The authors compared the effectiveness and cost measures of a ambulant psychiatric rehabilitation program on patients for the period of up to 12 months after discharge to the period of 12 months before admission to the intervention program based on self-reported catamnesis questionnaires using suitable statistical analyses in Vienna, Austria. The most important finding was that such an prevention program was especially beneficial for rehabilitation patients in earlier stages of psychiatric diseases who were still employed, indicating the need for early intervention in mental disorder.

To summarize, policy maker involvement in the development of applied OR models (e.g., Shetaban et al. 2020, Capan et al. 2017, Hulshof et al. 2012), behavioral OR (e.g., Brailsford et al. 2020b; Kunc et al. 2020; Donohue et al. 2018), hybrid OR modelling techniques (e.g., Brailsford et al. 2019; Xu et al. 2015), machine learning and data mining approaches (e.g., Ben-Israel et al. 2020; Malik et al. 2018), and sound prevention strategies (e.g., Silal 2021, Kong and Zhang 2018) proved the topics of highest importance for future OR applications in the field of strategic health care management.

## Health economic applications

Health economics uses various quantitative methods to investigate a wide variety of economic issues in the health sector. The papers presented in the health economics sessions at the ORAHS 2020 conference reflected this diversity.

An important branch of research in health economics is the measurement of health preferences using discrete choice experiments (DCEs). The corresponding research results are used, for example, to assess supply and demand and the pricing of various health services. Kleij et al. (2017) provided a systematic literature review of DCEs in primary health care, while Larsen et al. (2021) analyzed the evolution of DCEs, among others, for designing mental health services for patients and providers. Collacott et al. (2021) and Sugitani et al. (2020) reviewed the frequent use of DCEs in oncology to investigate patients' preferences. In a recent study, Michaels-Igbokwe et al. (2021) d, among others, on age-specific characteristics of designing, implementing, and analyzing DCEs in assessing the preferences of children and adolescents. From a methodological perspective, Bahrampour et al. (2020) provided an overview of the various methods to perform DCEs. Janssen et al. (2017) and Quaife et al. (2018) reviewed the (external) validity and reliability of DCEs. At the ORAHS 2020 conference, Zweifel (2020) discussed the use of DCEs in the context of costly innovation in health care. In the related paper (Zweifel, 2021), which is part of this issue, he seeks to justify using the economic criterion "provision of health care according to the preferences of current and potential patients" for guiding decisions concerning the adoption of costly innovation in health. For measuring these preferences, he highlights the use of DCEs to derive the willingness to pay (WTP) values that can be pitted against the extra cost of innovative therapies. The broad application across different medical disciplines and the constant methodological development illustrate the importance of preference measurement in supporting clinical and economic decisions (see, e.g., Khan et al., 2021).

Another relevant health economics research stream was devoted to designing optimal contracts among differently informed contractual partners with varying and potentially conflicting interests. Contract theory aims to improve the performance of a health (sub)system by incentivizing the efficient use of scarce resources on the supply and demand side while simultaneously guaranteeing the (sub)system's quality and effectiveness. There is vibrant literature on the effects of differently designed contracts on the supply, demand, quality, and accessibility of medical services, pharmaceuticals, and other health care products and services. Fainman and Kucukyazici (2020) provided a review of studies investigating the effect of financial incentives and payment mechanisms for service providers on accessibility, quality, efficiency, and integration and collaboration in health care. De Walque (2020) addressed the demand side by examining the effectiveness of various financial incentives to avoid unhealthy behavior. Antonanzas et al. (2019) offered an in-depth literature review of various risk-sharing contracts in healthcare settings. In their paper, the authors referred, among others, to an overview by Zaric et al. (2013). The latter reviewed theoretical papers on risk-sharing and examined the effects on the behavior of pharmaceutical companies and social welfare. In his keynote at the ORAHS 2020 conference, Zaric (2020) highlighted the importance of contract theory for health care policy. He presented principal-agent models on risk-sharing contracts in the pharmaceutical sector and gain-sharing agreements in the hospital sector. He also provided insight into the effects of incentive payments for Canadian doctors to provide after-hours primary care on hospital emergency department utilization. Megiddo et al. (2020) illustrated an extension to current health technology assessment methods that incorporate externalities associated with technological innovations. Their model enables the assessment of the societal value of innovative technology, which is considered an essential element in price negotiations. Barlow et al. (2020) discussed a UK pilot program that decomposed total payment for an antibiotic into a fixed lump sum and a sales-related component in a related presentation. The authors proposed a mathematical model that explicitly considered a flat and volume-based compensation component to estimate the societal impact of such a subscription payment system.

The need to use scarce resources efficiently while simultaneously guaranteeing and improving health services provision effectiveness has also fueled considerable interest in applying multi-objective optimization models, control theory, and performance measurement methods in the health sector. In their ORAHS 2020 presentation, Neuvonen and Dillon (2020) proposed decision programming to optimize a screening program for the early detection of colon cancer. With the mathematical model developed by the authors, they optimize the cut-off level of a specific screening program in a particular target population by minimizing direct costs and maximizing the reduction of incidence and mortality rates. Freiberger et al. (2020) presented a sophisticated dynamic optimal control framework that considers stochastic health shocks in life-cycle models that enabled the authors to obtain analytic pre- and post-shock expressions for consumption and health investment profiles. Sommersguter-Reichmann (2020) concentrated on the incorporation of quality in nonparametric frontier efficiency studies, which have attracted considerable attention since the seminal works of Farrell (1957), Charnes et al. (1978), as well as Färe and Knox Lovell (1978). The extensive bibliographies (e.g., Seiford 1996; Emrouznejad and Yang 2018), general and health-related reviews (e.g., Peykani et al., 2021, Mergoni and Witte 2021, Hollingsworth 2008, Cantor and Poh 2017, O'Neill et al. 2008, Kohl et al. 2019) and special issues (e.g., Emrouznejad et al. 2019; Emrouznejad 2014; Jablonsky et al. 2018) underpin the dynamic publication activity in this field. The author found that many studies assume that structural quality affects inefficiency distribution. At the same time, they presume that process and outcome quality predominantly impact the efficiency frontier (Sommersguter-Reichmann, 2021).

## Health care workforce scheduling care

At the ORAHS 2020 (https://orahs2020.univie.ac.at/), planning of home health care services as well as outpatient chemotherapy planning and clinical rostering constituted important OR application areas in health care.

### Planning of home health care services

Home health care (HHC) services are of vital importance for the health care system of many countries (Rest and Hirsch 2021). The number of people requiring HHC services is increasing in industrialized countries due to demographic transformation, changes in the family structure, the trend to grow old at home, or the usually lower cost than intramural care. Several recent literature reviews on HHC as Fikar and Hirsch (2017), Hirsch (2017), Cissé et al. (2017), Grieco et al. (2020), or Di Mascolo et al. (2021) give a comprehensive overview on this topic.

At the ORAHS 2020 some presentations dealt with HHC planning, e.g., Ramalhinho Lourenco et al. (2020), who propose models to optimize integrated HHC and home social care, and evaluate the cost and service quality impact of introducing synchronization in the whole system, or Salman et al. (2020), who focus on dynamic prioritized HHC routing and scheduling. The paper “Insights and decision support for home health care services in times of disasters” by Rest and Hirsch (2021) studies the impacts of various disasters (i.e., epidemics, blackouts, heatwaves, and floods) on the HHC system, using the concept of Causal-Loop-Diagrams. DSS is presented and applied to real-world data from a HHC service provider in Vienna, in order to numerically analyze the impacts of the COVID-19 pandemic that hit Austria in spring 2020. The DSS is based on a Tabu Search metaheuristic that specifically aims to deal with the peculiarities of urban regions. The impact of disasters on HHC planning has been covered in just a few papers as Trautsamwieser et al. (2011), Rest et al. (2012), or Rest and Hirsch (2015) before. Rest and Hirsch (2021) also provide solutions, if HHC nurses use time-dependent public transport (i.e., bus, tram, train, or underground) instead of an individual transport mode as car or bike. This has only been highlighted in a few preceding journal papers as Hiermann et al. (2015) or Rest and Hirsch (2016). Moreover, it is important to consider sustainability issues in HHC planning (Voegl and Hirsch 2018). The presentation of Reyes et al. (2020) considered an integrated mobility concept for HHC services and not time-critical patients, who need to be transported to medical facilities, in order to reduce driving distances of the employed vehicles and to support the use of more environment friendly transport modes as public transport.

### Outpatient chemotherapy planning and clinicians rostering

The paper "Master chemotherapy planning and clinicians rostering in a hospital outpatient cancer centre" by Carello et al. (2021), deals with a highly relevant and fairly complicated real-world tactical level planning problem. The problem addressed arises in outpatient cancer centers where different pathologies and oncologist specialties share the main resources. The problem aims at assigning consultation rooms and days to the treated pathologies and setting the medical agendas. Different criteria are considered. The problem is formulated as a multi-objective lexicographic optimization model and solved by sequentially optimizing a sequence of MIP models. The paper shows that the different stakeholders’ perspectives can be efficiently optimized and that a rolling horizon approach can be fruitfully applied over a one-year planning horizon. The practical orientation of the research is reinforced by the use of real data and the cooperation of hospital managers and oncologists. Carello et al. (2021) mention that the literature on outpatient chemotherapy activities is sparse compared to other areas of application with similar features, such as multi-appointment scheduling in healthcare (Marynissen et al. 2019). A literature review on operations research techniques applied throughout cancer care services can be found in Saville et al. (2019). Moreover, the review paper of Lamé (2016) provides an overview on outpatient chemotherapy planning.

At the ORAHS 2020, Penn (2020) gave a talk on the prisoner’s dilemma in healthcare scheduling based on data for chemotherapies. A dedicated session on chemotherapy and radiotherapy scheduling at the ORAHS 2020 included presentations of Robbes et al. (2020) on the production of chemotherapies, of Duenas et al. (2020) on multicriteria decision analysis modelling in an outpatient chemotherapy service, and of Aringhieri et al. (2020a,b) on online algorithms for the radiotherapy patient scheduling problem. A recent paper by Braune et al. (2021) presents a model for planning appointment times for radiotherapy treatments with uncertain activity durations, several treatment rooms, and required preparation as well as exiting phases for each patient.

## Health care operations management

Health care operations with the topics health care logistics as well as disease modelling constitute important OR application areas in health care covered by the ORAHS 2020 program (https://orahs2020.univie.ac.at/).

### Health care logistics

Health care logistics addresses the efficient planning, scheduling, and control of different flows in the delivery of health care services. Such flows are related to patients, materials, and information. The use of OR plays a crucial role when managers want to optimize processes considering a patient centered approach, that is not only taking into account the economic efficiency but also considering the quality of care and patient satisfaction. Health care logistics covers a broad area of health services such as the emergency medical services (e.g., Aringhieri et al. 2017; Reuter-Oppermann et al. 2017), health facilities (e.g., Ahmadi-Javid et al. 2017), operating room planning (e.g., Samudra et al. 2016; Zhu et al. 2019), appointment scheduling (e.g., Marynissen et al., 2019), hospital management (Abe et al. 2016a,b,c), and disaster management (e.g., Özdamar et al. 2015; Farahani et al. 2020).

At the ORAHS 2020 more than six sessions covered the broad area of the health care logistics. Hosteins et al. (2020) presented a talk on bed management, an emerging topic in health care. Beds are an essential resource, which follows the patients, and must be adequately managed. Stock-outs could lead to severe consequences: delays, redirections or procedure cancellations. Therefore, reliable and robust bed management is fundamental for well-performing hospitals. Landa et al. (2018) studied how to improve the bed capacity planning and its coordination with the management of emergency and elective patient admissions. Kayis et al. (2020) studied the integrated scheduling of ORs and sterilization of reusable medical devices under stochastic surgery durations with the possibility of dynamic rescheduling, if needed, during the day. Dynamic rescheduling or real time management of operating rooms is new and promising area of research as reported by Duma et al. (2015, 2018).

The aim of the paper "Fairness in ambulance routing for post disaster management" by Aringhieri et al. (2021) is to find the best ambulance tours to transport the patients during a disaster in relief operations while considering fairness and equity to deliver services to patients in balance. The problem is formulated as a new variant of the team orienteering problem with hierarchical objectives to address also the efficiency issue. A new hybrid algorithm based on a machine learning and neighbourhood search has been developed. An extensive quantitative analysis proved the capability of the algorithm allowing also a comparison between the fair solution and the system optimum.

### Disease policy modelling

The use of quantitative models can show how (infectious) disease progress over time and helping the development of public health interventions and policies such as mass vaccination. Children vaccination programmes have been discussed in two talks at the ORAHS 2020: the first one presented a rule-based digital vaccination decision support for child immigrants immunization coordination in Sweden (Steen et al. 2020) while the second presented a cross-sectional survey for childhood vaccination in Viennesse primary schools (Rauner et al. 2020).

The paper "Hospital preparedness during epidemics using simulation. The case of COVID-19", by Garcia de Vicuña et al. (2021) proposes a discrete event simulation model to support decision-making for the planning of hospital resource needs during pandemic waves. It also reports how it was employed on a daily basis to inform logistic health authorities and the lessons learnt from this successful application.

Together with two other talks, this paper was part of the special session "Covid-19 Policy Modelling" at the ORAHS 2020, which was held not in parallel with other sessions. Rutherford et al. (2020) developed a two-stage queue network model to support planning requirements for access to mechanical ventilators by both COVID-19 and non-COVID-19 patients in British Columbia, Canada. Duma et al. (2020) presented a new problem called the daily swab test collection problem, which consists in organizing the daily collection of swab tests reaching the house of the contact(s) of a positive case detected the day(s) before through a digital contact tracing system.

## Emergency services

Providing emergency care is a challenging task for all healthcare systems worldwide and the COVID-19 pandemic made it even more complex. In many countries, emergency medical services (EMS) care for emergency patients on the scene of the incident. While the Franco-German system emphasise the treatment at the scene, the Anglo-American system favours a fast transport to the hospital (Reuter-Oppermann et al. 2017). In hospitals, emergency departments (ED) are the gateway for all emergency patients seeking care, either when transported by an ambulance or arriving as walk-ins. At the ORAHS 2020 conference, four sessions directly targeted emergency services and the presentations are summarised in the following three subsections (https://orahs2020.univie.ac.at/).

### Emergency medical services

Within EMS logistics, many different planning problems occur that can be addressed by operations research methods. These include locating ambulances and bases, workforce planning of paramedics and emergency doctors, dispatching ambulances or even helicopters or relocating ambulances during the day. A description of the planning problems as well as an overview of existing publications can be found, for example, in the reviews by Reuter-Oppermann et al. (2017) or Aringhieri et al. (2017).

During ORAHS 2020, four talks addressed different EMS topics. Pieter van den Berg (2020) discussed and analyzed modelling flaws in ambulance location models that use busy fractions, as for example the MEXCLP (Daskin, 1983). The dispatching of ambulances was addressed by Carvalho et al. (2020) as well as Olave-Rojas and Nickel (2020). Worthington et al. (2020) investigated “optimal” service hours for helicopters and rapid response vehicles in the UK.

This issue contains one EMS-related paper by Matinrad and Reuter-Oppermann (2021) entitled “A review on initiatives for the management of daily medical emergencies prior to the arrival of emergency medical services “. The paper presents a review of the studies that are focused on the use of new types of resources, such as volunteers and drones, for daily medical emergency responses. These resources are used in daily medical emergencies if they can arrive earlier than emergency medical services. This work includes a total of 258 papers published in operations research/operations management and medical journals and conference proceedings. The paper investigates these studies from technical, logistical, and medical perspectives and gives an application-based and methodological overview.

### Emergency departments

In order to provide the best possible care for emergency patients, it is not sufficient to analyse and improve the EMS and EDs individually. Instead, a system’s perspective is necessary and the complete rescue chain should be addressed. In countries like Canada or the UK, for example, ambulances often queue in front of the ED due to overcrowding, leading to very long turnaround times (Li et al. 2019). At ORAHS 2020, Reuter-Oppermann and Wolff (2020b) presented the design of an emergency navigator that supports the EMS in assigning emergency patients to hospitals (Reuter-Oppermann and Wolff 2020a).

Within ED related research, many publications apply simulation as their main methodology and reviews summarise the state of the art (e.g., Salmon et al. 2018; Furian et al. 2018). During ORAHS 2020, several speakers presented simulation models for emergency departments (Roma et al. 2020; Fabbri et al. 2020; Vanbrabant et al. 2020). Mayorga and Nambiar (2020) proposed a queuing model to study and improve the workload of nurses and the patients’ lengths of stay. Bijvank and Lee (2020) discussed the integration of care complexity levels into patient triage systems used in Canadian EDs.

### Mass casualty incidents

Mass casualty incidents impose major challenges for EMS systems with treating many patients at the scene and transporting them to hospitals as well as for hospitals with many patients arriving at the same time in the ED. During the ORAHS 2020 conference, two talks specifically addressed mass casualty incidents. Li et al. (2020) discussed index policies for resource scheduling in emergency response. Christina Bartenschlager (2020) analyzed and compared existing and new triage algorithms for mass casualty incidents.

## References

[CR1] Abe TK, Beamon BM, Storch RL, Agus J (2016). Operations research applications in hospital operations: part I. IIE Trans Healthcare Syst Eng.

[CR2] Abe TK, Beamon BM, Storch RL, Agus J (2016). Operations research applications in hospital operations: part II. IIE Trans Healthcare Syst Eng.

[CR3] Abe TK, Beamon BM, Storch RL, Agus J (2016). Operations research applications in hospital operations: part III. IIE Trans Healthcare Syst Eng.

[CR4] Ahmadi-Javid A, Seyedi P, Syam SS (2017). A survey of healthcare facility location. Comput Oper Res.

[CR5] Amiri MM, Kazemian M, Motaghed Z, Abdi Z (2021). Systematic review of factors determining health care expenditures. Health Policy Technol.

[CR6] Antonanzas F, Juárez-Castelló C, Lorente R, Rodríguez-Ibeas R (2019). The use of risk-sharing contracts in healthcare: theoretical and empirical assessments. Pharmacoeconomics.

[CR7] Aringhieri R, Tànfani E, Testi A (2013). Operations research for health care delivery. Comput Oper Res.

[CR8] Aringhieri R, Knight V, Smith H (2016). ESI XXXI-OR applied to health in a modern world. Oper Res Health Care.

[CR9] Aringhieri R, Knight V, Smith H (2016). ESI XXXI: OR applied to health in a modern world. Health Syst.

[CR10] Aringhieri R, Bruni ME, Khodaparasti S, van Essen JT (2017). Emergency medical services and beyond: Addressing new challenges through a wide literature review. Comput Oper Res.

[CR11] Aringhieri, R., Duma, D., Squillace, G. (2020a) Online Algorithms for the Radiotherapy Patient Scheduling Problem. Presentation at the ORAHS 2020, https://orahs2020.univie.ac.at/

[CR12] Aringhieri, R., Duma, D., Squillace, G. (2020b). Pattern-based online algorithms for a general patient-centred radiotherapy scheduling problem. In: Paper presented at the Springer Proceedings in Mathematics and Statistics, 316, 251–262. 10.1007/978-3-030-39694-7_20

[CR13] Aringhieri R, Duma D, Guastalla A (2021) Fairness in ambulance routing for post disaster management. Central Eur J Oper Res. 10.1007/s10100-021-00785-y 10.1007/s10100-021-00785-y PMC8545622 34720734

[CR14] Aspland, E., Harper, P., Gartner, D., Arruda, E., Palmer, G., Webb, P., Barrett-Lee, P. (2020). Sim.Pro.Flow—Automating Simulation Build. Presentation at the ORAHS 2020, https://orahs2020.univie.ac.at/

[CR15] Bahrampour M, Byrnes J, Norman R, Scuffham P, Downes M (2020). Discrete choice experiments to generate utility values for multi-attribute utility instruments: a systematic review of methods. Eur J Health Econ Health Econ Prevent Care.

[CR16] Barlow, E., Morton, A., Megiddo, I., Colson, A. (2020) Investigating a Subscription Payment Model for Antibiotic Purchasing. Presentation at the ORAHS 2020, https://orahs2020.univie.ac.at/

[CR17] Barrera Ferro, D., Brailsford, S., Smith, H., Bayer, S. (2020). Should I Stay or ShouldI go? Understanding Noshow Behaviour Among Low-income Patients in Bogotá, Colombia. Presentation at the ORAHS 2020, https://orahs2020.univie.ac.at/

[CR18] Bartenschlager, C. (2020). *Triage Algorithms for Mass Casualty Incidents: Comparison, Analysis and New Systems*. Presentation at the ORAHS 2020, https://orahs2020.univie.ac.at/

[CR19] Ben-Israel D, Jacobs WB, Casha S, Lang S, Ryu WHA, de Lotbiniere-Bassett M, Cadotte DW (2020). The impact of machine learning on patient care: a systematic review. Artif Intell Med.

[CR20] Bijvank, M., Lee, S.-Y. (2020). *Characterization of Care Complexity in Emergency Departments*. Presentation at the ORAHS 2020, https://orahs2020.univie.ac.at/

[CR21] Bozorgmehr K, Roberts B, Razum O, Biddle L (2020). Health Policy and Systems Responses to Forced Migration.

[CR22] Braune R, Gutjahr WJ, Vogl P (2021). Stochastic radiotherapy appointment scheduling. CEJOR.

[CR23] Brailsford S, Harper P (2007). Editorial. J Oper Res Soc.

[CR24] Brailsford S, Harper P (2008). OR in health. Eur J Oper Res.

[CR25] Brailsford SC, Harper PR, Patel B, Pitt M (2009). An analysis of the academic literature on simulation and modelling in health care. J Simul.

[CR26] Brailsford S, Vissers J (2011). OR in healthcare: a European perspective. Eur J Oper Res.

[CR27] Brailsford S, Kozan E, Rauner MS (2012). Health care management. Flex Serv Manuf J.

[CR28] Brailsford S, Churilov L, Dangerfield B (2014). Discrete-event simulation and system dynamics for management decision making.

[CR29] Brailsford S, Eldabi T, Kunc M, Mustafee N, Osorio A (2019). Hybrid simulation modelling in operational research: a state-of-the-art review. Eur J Oper Res.

[CR30] Brailsford, S., Bayer, S., Connell, C., Klein, J. (2020a). Embedding OR/systems Modelling as Decision Support in Health Planning: Establishing a Community of Practice. Presentation at the ORAHS 2020, https://orahs2020.univie.ac.at/

[CR31] Brailsford, S., Carter, M., Harper, P., & Katsikopoulos, K. V. (2020b). Special issue on healthcare behavioural OR. J Oper Res Soc, 71(7), 1053–1054,

[CR32] Brandeau ML, Sainfort F, Pierskalla WP (2004). Operations Research and Health Care: a Handbook of Methods and Applications.

[CR33] Brandeau, M.L. (2020). *Predicting and Improving Patient-level Antibiotic Adherence*. Presentation at the ORAHS 2020, https://orahs2020.univie.ac.at/10.1007/s10729-020-09523-3 33017035

[CR34] Cantor V, Poh K (2017). Integrated analysis of healthcare efficiency: a systematic review. J Med Syst.

[CR35] Capan M, Khojandi A, Denton BT, Williams KD, Ayer T, Chhatwal J (2017). From data to improved decisions: operations research in healthcare delivery. Med Decis Mak.

[CR36] Carello G, Landa P, Tànfani E, Testi A (2021) Master chemotherapy planning and clinicians rostering in a hospital outpatient cancer centre. Central Eur J Oper Res. 10.1007/s10100-021-00786-x

[CR37] Carter MW, Price CC, Rabadi G (2018). Operations Research: a Practical Introduction.

[CR38] Carvalho, AS, Captivo ME, Marques I (2020) Integrating ambulance dispatching and relocation problems: the Portuguese case. Presentation at the ORAHS 2020, https://orahs2020.univie.ac.at/

[CR39] Cayırlı T, Günal MM, Güneş E, Örmeci L (2015). The 39th international conference of the EURO working group on operational research applied to health services: ORAHS 2013 special issue. Health Care Manag Sci.

[CR40] Chandra, D., Vipin B. (2020). *Contract Analysis for Rotavirus Vaccine Supply Chain in India*. Presentation at the ORAHS 2020, https://orahs2020.univie.ac.at/

[CR41] Charnes A, Cooper W, Rhodes E (1978). Measuring the efficiency of decision making units. Eur J Oper Res.

[CR42] Cissé M, Yalçındağ S, Kergosien Y, Şahin E, Lenté C, Matta A (2017). OR problems related to Home Health Care: a review of relevant routing and scheduling problems. Oper Res Health Care.

[CR43] Collacott H, Soekhai V, Thomas C, Brooks A, Brookes E, Lo R (2021). A systematic review of discrete choice experiments in oncology treatments. The Patient.

[CR44] Daskin MS (1983). A maximum expected covering location model: formulation, properties and heuristic solution. Transp Sci.

[CR45] Davies R, Bensley D (2005). Special issue: meeting health challenges with OR. J Oper Res Soc.

[CR46] Demiray, O., Gunes, E.D., Ormeci, E.L. (2020). *A Personalized Approach to the Allocation of Complex Care Delivery Under Parameter Uncertainty*. Presentation at the ORAHS 2020, https://orahs2020.univie.ac.at/

[CR47] De Walque D (2020). The use of financial incentives to prevent unhealthy behaviors: a review. Soc Sci Med.

[CR48] Dexter F, Marcon E, Xie X (2009). Operational research applied to health services 2007 special issue. Health Care Manag Sci.

[CR49] Di Mascolo M, Martinez C, Espinouse ML (2021). Routing and scheduling in home health care: a literature survey and bibliometric analysis. Comp Ind Eng.

[CR50] Donohue K, Katok E, Leider S (2018). The Handbook of Behavioral Operations.

[CR51] Duenas A, Glaize A, Di Martinelly C, Fagnot I (2020) MCDA Modelling in an Outpatient Chemotherapy Service. Presentation at the ORAHS 2020, https://orahs2020.univie.ac.at/

[CR52] Duma D, Aringhieri R (2015). An online optimization approach for the real time management of operating rooms. Oper Res Health Care.

[CR53] Duma D, Aringhieri R (2018). The real time management of operating rooms*. Operations Research Applications in Health Care Management*, volume 262 of *International Series in Operations Research & Management Science*, pages 55–79. Springer, 10.1007/978-3-319-65455-3_3

[CR54] Duma D, Aringhieri R, Bigharaz S, Druetto A, Grosso A (2020) Optimising the Daily Swab Test Collection to Identify New Cases of Covid-19. Presentation at the ORAHS 2020, https://orahs2020.univie.ac.at/

[CR55] Duque-Uribe, V., William Sarache, W., Valentina, Gutiérrez, E.V. (2020). *Sustainability in Hospital Supply Chain Management: Empirical Research in a Developing Country in Latin America*. Presentation at the ORAHS 2020, https://orahs2020.univie.ac.at/

[CR56] Emrouznejad A (2014). Advances in data envelopment analysis. Ann Oper Res.

[CR57] Emrouznejad A, Yang G (2018). A survey and analysis of the first 40 years of scholarly literature in DEA: 1978–2016. Socioecon Plann Sci.

[CR58] Emrouznejad A, Banker R, Neralić L (2019). Advances in data envelopment analysis: celebrating the 40th anniversary of DEA and the 100th anniversary of Professor Abraham Charnes' birthday. Eur J Oper Res.

[CR59] Fabbri, C., Bua, V., Malaguti, E., Monaci, M., Tubertini, P. (2020). A Simulation-based Dss for ED Process Management. Presentation at the ORAHS 2020, https://orahs2020.univie.ac.at/

[CR60] Färe R, Knox Lovell C (1978). Measuring the technical efficiency of production. J Econ Theory.

[CR61] Fainman E, Kucukyazici B (2020). Design of financial incentives and payment schemes in healthcare systems: a review. Soc Plann Sci.

[CR62] Farahani RZ, Lotfi MM, Baghaian A, Ruiz R, Rezapour S (2020). Mass casualty management in disaster scene: a systematic review of OR&MS research in humanitarian operations. Eur J Oper Res.

[CR63] Farrell M (1957). The measurement of productive efficiency. J R Statist Soc Ser A (general).

[CR64] Fikar C, Hirsch P (2017). Home health care routing and scheduling: a review. Comput Oper Res.

[CR65] Freiberger, M., Kuhn, M., Wrzacjek, S. (2020). *Prepare or React? Integrating Large Health Shocks Into Life-cycle Models.* Presentation at the ORAHS, https://orahs2020.univie.ac.at/

[CR66] Furian N, Neubacher D, O’Sullivan M, Walker C, Pizzera C (2018). GEDMod–Towards a generic toolkit for emergency department modeling. Simul Model Pract Theory.

[CR67] Garcia-Vicuña D, Esparza L, Mallor F (2021). Hospital preparedness during epidemics using simulation. The case of COVID-19. Central Eur J Oper Res.

[CR68] Grieco L, Utley M, Crowe S (2020). Operational research applied to decisions in home health care: a systematic literature review. J Oper Res Soc.

[CR69] Hans EW, Vliegen IM (2014). Editorial: Special issue of the 2012 conference of the EURO working group operational research applied to health services (ORAHS). Oper Res Health Care.

[CR70] Harper P, Baker R (2005). Editorial. IMA J Manag Math.

[CR71] Hiermann G, Prandtstetter M, Rendl A, Puchinger J, Raidl GR (2015). Metaheuristics for solving a multimodal home-healthcare scheduling problem. CEJOR.

[CR72] Hirsch P (2017). Recent planning approaches and mobility concepts for home health care services in Austria—A review. Bodenkultur.

[CR73] Hollingsworth B (2008). The measurement of efficiency and productivity of health care delivery. Health Econ.

[CR74] Hosteins, G., Larsen, A., Pacino, D., Sørup C. (2020). *Bed Management - Hybrid Simulation of a Bed Logistics Flow at a Danish Public Hospital*. Presentation at the ORAHS 2020, https://orahs2020.univie.ac.at/

[CR75] Hulshof PJ, Kortbeek N, Boucherie RJ, Hans EW, Bakker PJ (2012). Taxonomic classification of planning decisions in health care: a structured review of the state of the art in OR/MS. Health Systems.

[CR76] Jablonsky J, Emrouznejad A, Toloo M (2018). Special issue of data envelopment analysis. CEJOR.

[CR77] Janssen E, Marshall D, Hauber A, Bridges J (2017). Improving the quality of discrete-choice experiments in health: How can we assess validity and reliability?. Expert Rev Pharmacoecon Outcomes Res.

[CR78] Kahraman C, Topcu YI (2018). Operations research applications in health care management.

[CR79] Kayis, E., Coban, E., Farajkhah, S.K. (2020) *Stochastic Scheduling of Operating Rooms and Reusable Medical Devices Under Dynamic Rescheduling*. Presentation at the ORAHS, https://orahs2020.univie.ac.at/

[CR80] Karanikolos M, Heino P, McKee M, Stuckler D, Legido-Quigley H (2016). Effects of the global financial crisis on health in high-income OECD countries: a narrative review. Int J Health Serv.

[CR81] Kazakov, R., Howick, S., Morton, A. (2020). *Using a Hybrid System Dynamics and Agent-based Simulation to Evaluate the External Reference Pricing Regulation Effect on Access, Affordability and Availability*, Presentation at the ORAHS 2020, https://orahs2020.univie.ac.at/

[CR82] Khan I, Pintelon L, Martin H (2021). The application of multicriteria decision analysis methods in health care: a literature review. Med Decis Mak.

[CR83] Kleij K, Tangermann U, Amelung V, Krauth C (2017). Patients’ preferences for primary health care—a systematic literature review of discrete choice experiments. BMC Health Serv Res.

[CR84] Kohl S, Schoenfelder J, Fügener A, Brunner J (2019). The use of data envelopment analysis (DEA) in healthcare with a focus on hospitals. Health Care Manag Sci.

[CR85] Kunc M, Harper P, Katsikopoulos K (2020). A review of implementation of behavioural aspects in the application of OR in healthcare. J Oper Res Soc.

[CR86] Kong N, Zhang S (2018). Decision analytics and optimization in disease prevention and treatment.

[CR87] Lamé G, Jouini O, Stal-Le Cardinal J (2016). Outpatient chemotherapy planning: a literature review with insights from a case study. IIE Trans Healthcare Syst Eng.

[CR88] Landa P, Sonnessa M, Tànfani E, Testi A (2018). Multiobjective bed management considering emergency and elective patient flows. Int Trans Oper Res.

[CR89] Larsen A, Tele A, Kumar M (2021). Mental health service preferences of patients and providers: a scoping review of conjoint analysis and discrete choice experiments from global public health literature over the last 20 years (1999–2019). BMC Health Serv Res.

[CR90] Li D, Ding L, Connor S (2020) When to Switch? Index Policies for Resource Scheduling in Emergency Response. Presentation at the ORAHS 2020, https://orahs2020.univie.ac.at/

[CR91] Li M, Vanberkel P, Carter AJ (2019). A review on ambulance offload delay literature. Health Care Manag Sci.

[CR92] Makwana N (2020). Public health care system's preparedness to combat epidemics after natural disasters. J Family Med Primary Care.

[CR93] Malik MM, AbdallahAla’raj SM (2018). Data mining and predictive analytics applications for the delivery of healthcare services: a systematic literature review. Ann Oper Res.

[CR94] Mallor F, Brailsford S, Rauner M, Azcarate C (2018). Operational research applied to health services: finding better health-care decisions in new oceans of health data. Oper Res Health Care.

[CR95] Marynissen J, Demeulemeester E (2019). Literature review on multi-appointment scheduling problems in hospitals. Eur J Operat Res.

[CR1001] Masmoudi M, Jarboui B, Siarry P (eds) (2021) Operations research and simulation in healthcare. Springer International Publishing, Cham, Switzerland

[CR96] Matinrad N, Reuter-Oppermann M (2021). A review on initiatives for the management of daily medical emergencies prior to the arrival of emergency medical services. CEJOR.

[CR97] Mayorga M, Nambiar S (2020) Managing Emergency Department Patient Flow and Nurse Staffing Using Fluid Approximations for Multi-class Pooled Service Queues. Presentation at the ORAHS 2020, https://orahs2020.univie.ac.at/

[CR98] Megiddo I, Colson A, Barlow E, Morton A (2020) Methodology for Estimating the Private and Societal Values of Antimicrobials. Presentation at the ORAHS, https://orahs2020.univie.ac.at/

[CR99] Mergoni A, de Witte K (2021). Policy evaluation and efficiency: a systematic literature review. Int Trans Oper Res.

[CR100] Michaels-Igbokwe C, Currie G, Kennedy B, MacDonald K, Marshall D (2021). Methods for conducting stated preference research with children and adolescents in health: a scoping review of the application of discrete choice experiments. Patient.

[CR101] Morton A, Rauner M, Zaric G (2016). Special issue on healthcare, introduction to the special issue. EURO J Decis Processes.

[CR102] Morton A, Bish E, Megiddo I, Zhuang W, Aringhieri R, Brailsford S (2021). Introduction to the special issue: Management science in the fight against Covid-19. Health Care Manag Sci.

[CR103] Neuvonen L, Dillon M (2020) Multi-objective Optimization of a Colorectal Cancer Screening Programme*.* Presentation at the ORAHS, https://orahs2020.univie.ac.at/

[CR104] Özdamar L, Ertem MA (2015). Models, solutions and enabling technologies in humanitarian logistics. Eur J Oper Res.

[CR105] Olave-Rojas D, Nickel S (2020) The Challenge of Dispatching the Right Ambulance: a Simulation-optimization Approach. Presentation at the ORAHS 2020, https://orahs2020.univie.ac.at/

[CR106] Oliveira M, Freitas L, Vieira A, Dimitrovová K, Bana e Costa C (2020) Designing Delphi Knowledge Construction Processes to Enable OR Behavioural Research and Extend Stakeholders’ Analyses in Health Settings. Presentation at the ORAHS 2020, https://orahs2020.univie.ac.at/

[CR107] O’Neill L, Rauner M, Heidenberger K, Kraus M (2008). A cross-national comparison and taxonomy of DEA-based hospital efficiency studies. Socioecon Plann Sci.

[CR108] Ozcan YA (2005). Quantitative Methods in Health Care Management: Techniques and Applications.

[CR109] Penn, M. (2020) *The Prisoner’s Dilemma in Healthcare Scheduling*. Presentation at the ORAHS 2020, https://orahs2020.univie.ac.at/

[CR110] Peykani P, Hosseinzadeh Lotfi F, Sadjadi S, Ebrahimnejad A, Mohammadi E (2021). Fuzzy chance-constrained data envelopment analysis: a structured literature review, current trends, and future directions. Fuzzy Optim Decis Making.

[CR111] Potgieter, L, Matter, D (2020) Resource Delivery and Allocation Strategies for Epidemic Response in a Rural Context. Presentation at the ORAHS 2020, https://orahs2020.univie.ac.at/

[CR112] Quaife M, Terris-Prestholt F, Di Tanna G, Vickerman P (2018). How well do discrete choice experiments predict health choices? A systematic review and meta-analysis of external validity. Eur J Health Econom Health Econom Prevent Care.

[CR113] Rais A, Viana A (2011). Operations research in healthcare: a survey. Int Trans Oper Res.

[CR114] Rauner MS, Vissers JM (2003). OR applied to health services: planning for the future with scarce resources. Eur J Oper Res.

[CR115] Rauner MS, Behrens DA, Wild C (2005). Quantitative decision support for health services. CEJOR.

[CR116] Rauner, M., Schnedlitz, S., Blaschke, S. (2020). Childhood Vaccination in Vienna: A Cross-sectional Survey Conducted in 4th Grades of Public Primary Schools. Presentation at the ORAHS 2020, https://orahs2020.univie.ac.at/

[CR117] Ramalhinho Lourenco, H., de Armas, J., Lima, M. (2020). The Impact in Synchronization in Home Health and Social Care Services. Presentation at the ORAHS 2020, https://orahs2020.univie.ac.at/

[CR118] Reyes, L., Voegl, J., Hirsch, P. (2020). Evaluation of an Integrated Mobility Concept for Home Care Staff and Ambulant Patients. Presentation at the ORAHS 2020, https://orahs2020.univie.ac.at/

[CR119] Rest KD, Trautsamwieser A, Hirsch P (2012). Trends and risks in home health care. J Humanit Logist Supply Chain Manage.

[CR120] Rest KD, Hirsch P (2015). Supporting urban home health care in daily business and times of disasters. IFAC-PapersOnLine.

[CR121] Rest KD, Hirsch P (2016). Daily scheduling of home health care services using time-dependent public transport. Flex Serv Manuf J.

[CR122] Rest KD, Hirsch P (2021). Insights and decision support for home health care services in times of disasters. CEJOR.

[CR123] Reuter-Oppermann M, van den Berg PL, Vile JL (2017). Logistics for emergency medical service systems. Health Systems.

[CR124] Reuter-Oppermann, M., Wolff, C. (2020a). *Enabling Customer-Centric Emergency Logistics Through Systems Thinking*. In European Conference on Information Systems (ECIS)-Marrakech, Marocco, June 15–17, 2020.

[CR125] Reuter-Oppermann M, Wolff C (2020b) Designing an Emergency Navigator for Assigning Emergency Patients to Hospitals. Presentation at the ORAHS 2020, https://orahs2020.univie.ac.at/

[CR126] Robbes A Kergosien Y, Billaut JC (2020) Chemotherapy Production Bilevel Problem: Cost Versus Delay. Presentation at the ORAHS 2020, https://orahs2020.univie.ac.at/

[CR127] Roma M, De Santis A, De Vito L, Giovannelli T, Lucidi S, Messedaglia M, Petitti, F., Romano, F. (2020). A Simulation-Optimization Approach for Parameter Calibration of Emergency Department Discrete Event Simulation Model. Presentation at the ORAHS 2020, https://orahs2020.univie.ac.at/

[CR128] Rouzet D, Sánchez AC, Renault T, Roehn O (2019) Fiscal challenges and inclusive growth in ageing societies, OECD Economic Policy Papers, No. 27, OECD Publishing, Paris, 10.1787/c553d8d2-en.

[CR129] Royston, G (2016) One hundred years of Operational Research in Health—UK 1948–2048. In Operational Research for Emergency Planning in Healthcare: Volume 2 (pp. 316–338). Palgrave Macmillan, London, 10.1007/978-1-137-57328-5_14

[CR130] Rutherford, A., Zimmerman, S., Dodek, P., Norena, M., van der Waall, A. (2020). A Queue Network Model of Ventilator Access During the COVID-19 Epidemic. Presentation at the ORAHS 2020, https://orahs2020.univie.ac.at/

[CR131] Salman, S., Cinar, A., Araz, O. (2020). Dynamic Prioritized Home Healthcare Routing and Scheduling in Emergencies and Routine Services. Presentation at the ORAHS 2020, https://orahs2020.univie.ac.at/

[CR132] Salmon A, Rachuba S, Briscoe S, Pitt M (2018). A structured literature review of simulation modelling applied to emergency departments: current patterns and emerging trends. Operat Res Health Care.

[CR133] Samudra M, Van Riet C, Demeulemeester E, Cardoen B, Vansteenkiste N, Rademakers FE (2016). Scheduling operating rooms: achievements, challenges and pitfalls. J Sched.

[CR134] Santamaria-Acevedo, G. (2020). Improvement of Patients Outcome Through the Optimization of Pre-surgical Times and Length of Stay (LOS). Presentation at the ORAHS 2020, https://orahs2020.univie.ac.at/

[CR135] Saville CE, Smith HK, Bijak K (2019). Operational research techniques applied throughout cancer care services: a review. Health Syst.

[CR136] Schosser A, Senft B, Rauner MS (2021). The benefit of an ambulant psychiatric rehabilitation program in Vienna, Austria: an uncontrolled repeated measures study. CEJOR.

[CR137] Seiford L (1996). Data envelopment analysis: the evolution of the state of the art (1978–1995). J Prod Anal.

[CR138] Sharma A, Borah SB, Moses AC (2021). Responses to COVID-19: the role of governance, healthcare infrastructure, and learning from past pandemics. J Bus Res.

[CR139] Shetaban S, Seyyed Esfahani MM, Saghaei A, Ahmadi A (2020). Operations research and health systems: a literature review. J Ind Eng Manage Stud.

[CR140] Silal SP (2021). Operational research: a multidisciplinary approach for the management of infectious disease in a global context. Eur J Oper Res.

[CR141] Sommersguter-Reichmann, M. (2020). *Frontier Efficiency Studies in Healthcare and Quality: A Review.* Presentation at the ORAHS, https://orahs2020.univie.ac.at/

[CR142] Sommersguter-Reichmann M (2021). Health care quality in nonparametric efficiency studies: a review. CEJOR.

[CR143] Steen, O., Holmberg, N. (2020). A Rule-based Digital Vaccination Decision Support for Child Immigrants Immunization Coordination in Sweden—The VacSam Digital Service. Presentation at the ORAHS 2020, https://orahs2020.univie.ac.at/

[CR144] Sugitani Y, Sugitani N, Ono S (2020). Quantitative preferences for lung cancer treatment from the patients’ perspective: a systematic review. The Patient.

[CR145] Trautsamwieser A, Gronalt M, Hirsch P (2011). Securing home health care in times of natural disasters. Or Spectrum.

[CR146] Tuson, M., Harper, P., Gartner, D. (2020). *Evaluating the Effectiveness and Efficiency of a Waleswide Public Health Initiative*. Presentation at the ORAHS 2020, https://orahs2020.univie.ac.at/

[CR147] Vanbrabant, L., Braekers, K. Ramaekers, K. (2020). Analysing the Impact of Workload-based Patient to Physician Assignment on Emergency Department Performance by Use of Discrete-event Simulation. Presentation at the ORAHS 2020, https://orahs2020.univie.ac.at/

[CR148] Van den Berg, P. (2020). *Overcoming Modeling Flaws in Ambulance Location Models*. Presentation at the ORAHS 2020, https://orahs2020.univie.ac.at/

[CR149] Vasilakis, C., Wood, R. (2020). Analyst-driven Development of an Open-source Simulation Tool to Address Poor Uptake of O.R. in Healthcare. Presentation at the ORAHS 2020, https://orahs2020.univie.ac.at/

[CR150] Vissers J, Beech R (2005). Health operations management: patient flow logistics in health care.

[CR151] Voegl, J., Hirsch, P. (2018.) The trade-off between the three columns of sustainability: A case study from the home service industry. In: Sustainable Transportation and Smart Logistics: Decision-Making Models and Solutions, pp. 467–486. 10.1016/B978-0-12-814242-4.00018-1

[CR152] WeberBlazewicz GWJ, Rauner M, Türkay M (2014). Recent advances in computational biology, bioinformatics, medicine, and healthcare by modern OR. CEJOR.

[CR153] Watts N, Amann M, Arnell N, Ayeb-Karlsson S, Beagley J, Belesova K (2020). The 2020 report of The Lancet Countdown on health and climate change: responding to converging crises. The Lancet.

[CR154] Worthington, D., Brooks, R., Morgan, L., Briggs, D. (2020). An Investigation Into ’Optimal Service Hours’ for the Operation of Response Vehicles for the North West Air Ambulance Charity (NWAA). Presentation at the ORAHS 2020, https://orahs2020.univie.ac.at/

[CR155] Xie X, Gallivan S, Guinet A, Rauner M (2010). Operational research applied to health services: a special volume dedicated to the international conference ORAHS’2007. Ann Oper Res.

[CR156] Xu J, Huang E, Chen CH, Lee LH (2015). Simulation optimization: a review and exploration in the new era of cloud computing and big data. Asia-Pacific J Oper Res.

[CR157] Zaric GS (2013). operations research and health care policy.

[CR158] Zaric G, Zhang H, Mahjoub R, Zaric G (2013). Modeling risk sharing agreements and patient access schemes. Operations Research and Health Care Policy.

[CR159] Zaric, G (2020) Incentives and Coordination in Healthcare*.* Presentation at the ORAHS, https://orahs2020.univie.ac.at/

[CR160] Zhu S, Fan W, Yang S, Pei J, Pardalos PM (2019). Operating room planning and surgical case scheduling: a review of literature. J Comb Optim.

[CR161] Zweifel, P (2020) Preference measurement in health using experiments. Presentation at the ORAHS, https://orahs2020.univie.ac.at/

[CR162] Zweifel P (2021). Preference measurement in health using experiments. CEJOR.

